# Withdrawn: The LncRNA H19/microRNA‐29b‐3p/HMGB1 signaling axis contributes to the regulation of lung cancer cell growth

**DOI:** 10.1002/2211-5463.13103

**Published:** 2022-01-11

**Withdrawn:** ‘The LncRNA H19/microRNA‐29b‐3p/HMGB1 signaling axis contributes to the regulation of lung cancer cell growth’, Xiaojun Zhong, Congkai Zhang, Yunlian Diao, Shu Liao, Qingyuan Ling, Zhuoxian Zhang,Jiuhong Zhong, Ping Long. The above article, published online on 2nd February 2021 in Wiley Online Library (wileyonlinelibrary.com) as an Accepted Article, has been withdrawn by agreement between the journal Editor‐in‐Chief Miguel De la Rosa, the FEBS Press Editorial Office, and John Wiley and Sons Ltd. The withdrawal has been agreed because the authors are not responding to requests to finalize their article for publication in the journal as the Version of Record. https://doi.org/10.1002/2211‐5463.13103

